# The combined effect of cranial-nerve non-invasive neuromodulation with high-intensity physiotherapy on gait and balance in a patient with cerebellar degeneration: a case report

**DOI:** 10.1186/s40673-018-0084-z

**Published:** 2018-03-05

**Authors:** Andisheh Bastani, L. Eduardo Cofré Lizama, Maryam Zoghi, Grant Blashki, Stephen Davis, Andrew H. Kaye, Fary Khan, Mary P. Galea

**Affiliations:** 1 Department of Rehabilitation Medicine, Royal Melbourne Hospital, RMH-Royal Park Campus, 34-54 Poplar Rd Parkville, Melbourne, VIC 3052 Australia; 2 Australian Rehabilitation Research Centre (ARRC), Royal Melbourne Hospital, Melbourne, Australia; 3 Department of Rehabilitation, Nutrition and Sport, La Trobe University, Bundoora, Melbourne, Australia; 4 The Nossal Institute for Global Health, The University of Melbourne, Melbourne, Australia; 5 Department of Medicine and Neurology, Melbourne Brain Centre at the Royal Melbourne Hospital, University of Melbourne, Melbourne, Australia; 6 Department of Surgery, The University of Melbourne, Melbourne, Australia

**Keywords:** Portable neuromodulation stimulator, Tongue stimulator, Neuroplasticity, Stroke

## Abstract

**Background:**

Cranial-nerve non-invasive neuromodulation (CN-NINM) using the portable neuromodulation stimulator (PoNS™^)^ device has been proposed as a novel adjuvant intervention to improve efficacy of gait and balance. This device modulates input and output signals during motor tasks which prompts neuroplastic changes. In this study, we investigated the efficacy of physiotherapy using the PoNS™ in a case with cerebellar degeneration.

**Case presentation:**

The PoNS™ was used during a high-intensity physiotherapy programme delivered over 2 weeks (2 × 1.5 h sessions daily). Clinical and instrumented gait and balance tests were applied pre- and post-intervention.

**Results:**

The patient improved in all tests without any adverse effects.

**Conclusion:**

This study showed the efficacy and feasibility of combined high-intensity physiotherapy and CN-NINM for gait and balance rehabilitation. Further studies should explore CN-NINM effects in larger and more diverse samples of neurological patients.

## Background

Gait and balance problems are common in most patients with neurological diseases, leading to high prevalence of falls, and considerable negative effects on mobility, function and quality of life. Despite strong evidence in regards to the effectiveness of physiotherapy interventions in improving gait and balance in patients with neurological disorders, there is still a need for optimisation of functional outcomes [1–4].

Neuromodulatory techniques such as repetitive transcranial magnetic stimulation (rTMS) and transcranial direct current stimulation (tDCS) have been utilized in different neurological populations to enhance rehabilitation outcomes, evidence shows mixed results of their effectiveness [1, 5, 6]. A novel approach using cranial-nerve non-invasive neuromodulation (CN-NINM) has been more recently applied in patients with multiple sclerosis, traumatic brain injury and stroke survivors, with positive results [7, 8]. Non-invasive stimulation of the facial and trigeminal nerves is delivered using electrodes embedded in a mouthpiece that rests on the tongue. The PoNS™ device is thought to modulate neural impulses to the brainstem and cerebellum, hence eliciting more targeted neuroplastic changes depending on the task performed during its use [9].

In this study we investigated the feasibility and efficacy of using the PoNS™ combined with high-intensity physiotherapy as a novel approach for improving gait and balance in a patient with cerebellar degeneration.

## Case presentation

A 55 years old woman who was suffering from a slow progressive spinocerebellar ataxia over the last 20 years participated in this study. Her last MRI showed cerebellar atrophy. She reported that she was a frequent faller due to her poor balance. She believed that one of the major contributors to her falls was having spasticity in her lower limbs.

Since the PoNS™ is not registered in Australia, it was used with approval from the Therapeutic Goods Administration (TGA) of Australia and the Melbourne Health Human Research Ethics Committee. The patient provided informed consent.

*Intervention* consisted of 2 physiotherapy sessions per day, 1.5 h per session (3 h between sessions) for 2 weeks (18 sessions in total plus pre- and post- intervention assessment sessions). Each session was structured in three blocks of 20 min, each focusing on gait performance, balance control and a relaxation/meditation period in the same order. During each block the patient used the PoNS™ device at a comfortable intensity which could be adjusted by the patient or therapist. At least 5 min of rest was given between stimulation blocks. Since the PoNS™ mouthpiece sits on the tongue, the patient was allowed to remove the device in case of excessive salivation to swallow or clean up.

*Clinical assessment* of gait and balance was performed using the miniBEST, which is a 14-item test scored on a 3-level ordinal scale with a maximum of 28 points. It focuses on anticipatory postural adjustments, reactive postural control, sensory orientation and gait [10]. The Depression Anxiety Stress Scale (DASS) and cognitive function using the CogLog were also used to assess negative emotional states and memory functioning and executive skills, respectively [11, 12].

*Gait assessment* was performed using a set of 4 inertial sensors sampling at 128 Hz (APDM, Portland, OR, USA) placed at the ankles, sacrum and chest, from which spatiotemporal and ranges of motion (RoM) measures were obtained over 40 gait cycles. Included walking measures were: speed (pace of walking), stride length (distance between any two successive points of heel contact of the same foot), cadence (number of steps per unit time), trunk RoM (the full movement potential of trunk) and percentages of stance (when the foot remains in contact with the ground), swing (when the reference foot is not in contact with the ground and swings in the air) and double support (both feet are in contact with the ground) time.

*Posturography* was used to assess balance using the same APDM system during double support (DS), tandem (TS) and single-leg (SL) stance for 30 s.

### Clinical assessments

The patient completed the 2-week intervention with no adverse effects related to the intensity of the intervention or the use of PoNS device. In the miniBEST, improvements were calculated as the percentage of the possible improvement points, that is: post- minus pre-intervention score as a percentage of 28 (maximum score) minus baseline score. Her score increased by 12 of 19 potential improvement points (63%). Interestingly, most of the improvements occurred in the dynamic (gait) section of the test, which assesses gait speed, turns and obstacle negotiation. She also showed considerable improvement in the anticipatory tasks, being able to stand from a chair independently, rising on toes for more than 3 s, and single-leg standing, as well as in the reactive balance tasks (Table 1). In addition, the patient showed a decrease in DASS and CogLog scores.

**Table 1:** Pre and Post intervention spatiotemporal measurements of balance and gait

	Pre		Post	
	mean	sd	mean	sd
Stride Length (%stature)	79.16	3.04	78.61	2.71
Stride Length SI (m)	1.23	0.08	1.22	0.08
Stride Velocity (%stature/s)	79.5	3.64	79.93	2.31
Stride Velocity SI (m/s)	1.24	0.07	1.24	0.05
Cadence (steps/min)	120.55	4.41	122.07	3.65
Gait Cycle Time (s)	1	0.04	0.98	0.03
Double support (%GC)	25.26	2.58	25.11	1.85
Swing (%GC)	37.37	1.29	37.45	0.93
Stance (%GC)	62.63	1.29	62.55	0.93
RoM Shank (°)	72.52	3.86	72.69	1.28
RoM Knee (°)	65.61	6.27	62.94	3.33
Peak Shank Velocity (°/s)	408.48	22.67	417.07	20.22
Peak Arm Swing Velocity (°/s)	192.23	33.82	191.04	25.02
Stride Length Asym. (%)	2.68	2.39	1.7	1.35
Phase Difference (°)	185.47	4.14	184.83	3.7
Phase Coordination Index (%)	11.62	2.54	11.86	2.19

*Gait assessment* showed that most spatiotemporal measures remained similar after intervention with a slight increase in the cadence. However, greater changes were observed on arm (20% increases in RoM) and trunk motion (22% more horizontal and 22% less frontal RoM) during walking. Furthermore, in the symmetry measures, asymmetry of stride length and velocity (> 35%) was decreased (Table 1).

**Fig. 1:**
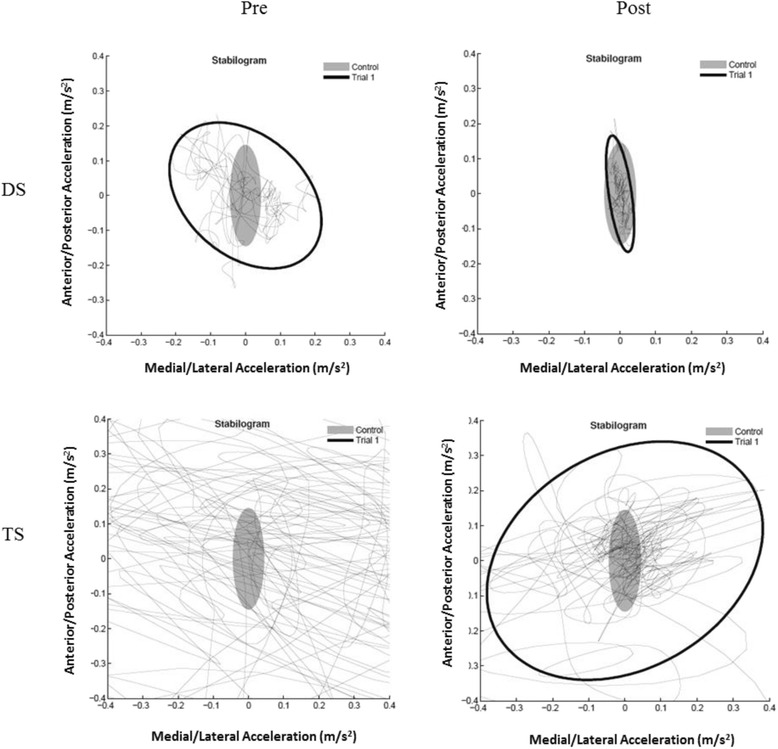
Pre and Post intervention posturographic measurements of balance during double support (DS) and tandem (TS) stance

*Posturographic* measures of balance showed large reductions in jerk, sway area and mean velocity for the DS and TS conditions (> 77%). Interestingly, mean velocity in the anteroposterior (AP) direction was increased (> 25%) but decreased in the mediolateral (ML) direction (> − 76%) for the same conditions (DS and TS) (Fig. 1). These changes were less pronounced in the SL conditions, which were the most challenging. Other non-linear measures of balance, e.g. mean frequency, also showed important reductions.

## Discussion and conclusions

Gait and balance impairments strongly affect independence and quality of life in patients with neurological disorders. Recent studies have shown that CN-NINM is effective in MS and stroke, but there is a need to explore its potential for rehabilitation of gait and balance in other neurological populations [7, 8]. This study explored the effect of combined high-intensity physiotherapy and CN-NINM on gait and balance in a patient with cerebellar degeneration. After the 2-week intervention, the patient achieved the maximum score in the reactive section of the miniBEST (6 points). Although we did not train the specific reactions assessed in the test, reactive balance responses may have been indirectly trained during “throw-and-catch” tasks and walking on unstable surfaces. It is well-known that reactive balance control is crucial with unexpected perturbations during daily-life (e.g. being pushed in busy places or on public transport). PoNS™ modulates both the afferent (i.e. feedback) and efferent (i.e. motor output) responses, which is essential for adequate balance responses. The patient showed substantial improvement in the anticipatory and dynamic sections of the miniBEST, which indicates that she has improved in controlling voluntary movements that also involves motor planning of more complex tasks (e.g. stepping over obstacles).

Moreover, we believe that the observed differences in pre- and post-intervention for the DASS and CogLog, cannot be directly linked to the intervention since this did not target emotional states or memory functioning. However, the decrease in depression score may be associated with the positive effects of increased physical activity experienced during 2 weeks activity, which was higher than usual as reported by the patient.

Walking speed [13] and stride velocity are important objective values of general health. These were improved in the patient after 2 weeks intervention by increasing her cadence rather than using a step lengthening strategy [14, 15]. The patient also spent less time in double support phase and greater time in single stance phases; although these changes were small, they support findings of a more stable walking after intervention as seen in the miniBEST dynamic section. With respect to kinematic changes, the patient increased transverse trunk rotation and decreased frontal trunk rotation and peak velocity, possibly as a more efficient walking strategy. She also improved in measures of gait asymmetry and coordination, which may be due to the intensive treadmill training used to improve and correct abnormal patterns.

Although balance was improved in all conditions and on most traditional measures (sway area, velocity, SD, and RMS), the patient had greater reductions of sway. Furthermore, she was able to perform SL standing with eyes closed for 30 s. Improvements in balance control after using the PoNS™ were also reported in MS patients when assessed under different sensory perturbation conditions [7].

Although physiotherapy is recommended as best practice for people with progressive ataxia, most research has involved single case studies [16]. A non-controlled trial [17] (*n* = 16) and randomised controlled trial with waitlist control [18] (*n* = 42) of 4 weeks of intensive coordination and balance training for patients with various types of cerebellar ataxia showed positive effects. Our patient underwent a much higher number of sessions of active physiotherapy time than that usually delivered to the patients with neurological disorders during standard rehabilitation [19]. We structured our physiotherapy intervention according to previous intervention programmes using the PoNS [9]. Although our results for this particular case are promising, we suggest a randomized controlled trial to determine whether the high-intensity physiotherapy or its combination with the CN-NINM is more effective for gait and balance rehabilitation in patients with cerebellar degeneration.

## Abbreviations

CN-NINM: Cranial-nerve non-invasive neuromodulation
DASS: Depression Anxiety Stress Scale
DS: Double support
PONS: Portable neuromodulation stimulator
rTMS: Repetitive transcranial magnetic stimulation
SL: Single-leg
tDCS: Transcranial direct current stimulation
TS: Tandem stance
